# Evaluation of *in-vitro* susceptibility of ß-lactam-resistant Gram-negative bacilli to ceftazidime-avibactam and ceftolozane-tazobactam from clinical samples of a general hospital in southern Brazil

**DOI:** 10.1590/0037-8682-0277-2022

**Published:** 2023-01-23

**Authors:** Thaisa Noceti Carvalho, Vanessa Cristine Kobs, Daniela Hille, Roseneide Campos Deglmann, Luiz Henrique Melo, Paulo Henrique Condeixa de França

**Affiliations:** 1 Universidade da Região de Joinville, Joinville, SC, Brasil.; 2 Dona Helena Hospital, Joinville, SC, Brasil.

**Keywords:** Antimicrobial resistance, Gram-negative bacilli, Ceftazidime-avibactam, Ceftolozane-tazobactam, *In vitro* activity, Genetic marker

## Abstract

**Background:**

The spread of carbapenemase- and extended-spectrum β-lactamase (ESBL)-producing gram-negative bacilli (GNB) represent a global public health threat that limits therapeutic options for hospitalized patients. This study aimed to evaluate the *in-vitro* susceptibility of β-lactam-resistant GNB to ceftazidime-avibactam (C/A) and ceftolozane-tazobactam (C/T), and investigate the molecular determinants of resistance.

**Methods:**

Overall, 101 clinical isolates of Enterobacterales and *Pseudomonas aeruginosa* collected from a general hospital in Brazil were analyzed. Susceptibility to the antimicrobial agents was evaluated using an automated method, and the minimum inhibitory concentrations (MIC50/90) of C/A and C/T were determined using Etest^®^. The β-lactamase-encoding genes were investigated using polymerase chain reaction.

**Results:**

High susceptibility to C/A and C/T was observed among ESBL-producing Enterobacterales (100% and 97.3% for CLSI and 83.8% for BRCAST, respectively) and carbapenem-resistant *P. aeruginosa* (92.3% and 87.2%, respectively). Carbapenemase-producing *Klebsiella pneumoniae* exhibited high resistance to C/T (80%- CLSI or 100%- BRCAST) but high susceptibility to C/A (93.4%). All carbapenem-resistant *K. pneumoniae* isolates were susceptible to C/A, whereas only one isolate was susceptible to C/T. Both antimicrobials were inactive against metallo-β-lactamase-producing *K. pneumoniae* isolates. Resistance genes were concomitantly identified in 44 (44.9%) isolates, with *bla*_CTX-M_ and *bla*_SHV_ being the most common.

**Conclusions:**

C/A and C/T were active against microorganisms with β-lactam-resistant phenotypes, except when resistance was mediated by metallo-β-lactamases. Most C/A- and C/T-resistant isolates concomitantly carried two or more β-lactamase-encoding genes (62.5% and 77.4%, respectively).

## INTRODUCTION

Significant clinical and economic impacts are often reported because of bacterial resistance, since long hospital stays and the empirical use of different antimicrobial agents increase healthcare costs, as well as morbidity, and mortality rates^1^. The rapid spread of carbapenemase- and extended-spectrum β-lactamase (ESBL)-producing gram-negative bacilli (GNB) represents an important threat to global public health^2^ ^,^ ^3^ and has limited the use of broad-spectrum cephalosporins and carbapenems in hospitalized patients^1^ ^,^ ^4^. 

In 2017, the World Health Organization (WHO) published a list of potentially critical multidrug-resistant microorganisms with global priority for the research and development of new antimicrobials, including carbapenem-resistant *Pseudomonas aeruginosa* and Enterobacterales resistant to carbapenems and third-generation cephalosporins^5^. A few antimicrobial agents have been developed in recent years to combat infections caused by multidrug-resistant GNB^6^ ^,^ ^7^. 

Ceftazidime-avibactam and ceftolozane-tazobactam were approved by the Food and Drug Administration (FDA) and the Brazilian Health Regulatory Agency (ANVISA) for the treatment of complicated intra-abdominal and urinary infections^8^ ^,^ ^9^. Ceftazidime-avibactam exerts *in-vitro* activity against clinical ESBL-producing isolates, including Ambler classes A (serine carbapenemases [KPC]), C (cephalosporinase-AmpC), and some class D enzymes (oxacillinases), but not metallo-β-lactamases (MβL)^9^ ^,^ ^10^. With the addition of avibactam, a β-lactamase inhibitor, ceftazidime tends to expand its activity against resistant strains^6^. Ceftolozane-tazobactam, which is currently approved for the treatment of hospital-acquired and mechanical ventilation-associated bacterial pneumonia^11^, is a combination of a fifth-generation cephalosporin and a known β-lactamase inhibitor. These agents together exert broad-spectrum activity against gram-negative bacteria, especially multidrug-resistant *P. aeruginosa* ^12^ ^-^ ^15^.

Although recently approved for clinical use and despite its proven efficacy against GNB, resistance to ceftazidime-avibactam and ceftolozane-tazobactam has been reported in several countries^16^. In this context, the present study evaluating the *in-vitro* activity of these antimicrobial agents, as well as genotypic resistance markers, is important for optimizing their use and will also contribute to the understanding of the current epidemiological scenario.

## METHODS

### Study characterization and selection of clinical isolates

This descriptive study focused on the phenotypic and molecular investigation of *P. aeruginosa* and Enterobacterales resistant to at least one carbapenem antibiotic or ESBL-producing antibiotic. The isolates were obtained sequentially from microbiological cultures of clinical samples collected from a general hospital in southern Brazil from January 2018. The clinical samples were subjected to routine procedures in the microbiology laboratory of the hospital for the identification of each microorganism, using the automated Microscan Walkaway Plus system (Beckman Coulter, USA) as well as Gram staining. 

### Phenotypic determination of antimicrobial susceptibility

The antimicrobial susceptibility profile was evaluated using the Kirby-Bauer disk diffusion method and the automated Microscan Walkaway Plus system (Beckman Coulter, USA) to determine the minimum inhibitory concentration (MIC) of each antimicrobial agent. Additionally, the MICs of ceftazidime-avibactam and ceftolozane-tazobactam were defined by a quantitative method using standardized Etest^®^ strips that contained an exponential concentration gradient. The concentration range used for both antimicrobials was 0.016/4-256/4 mg/L and the results were interpreted using the parameters of the Clinical and Laboratory Standards Institute (CLSI) and of the Brazilian Committee on Antimicrobial Susceptibility Testing (BRCAST).

The isolates were classified as ESBL producers based on the observation of a reduction in the inhibition halos for broad-spectrum β-lactams in the antimicrobial susceptibility test and double-disk synergy test. Isolates were classified as resistant to carbapenems (CR) when resistance to meropenem, ertapenem, or imipenem was identified. The phenotypic detection of carbapenemases was performed using the enzymatic blocking method described in ANVISA Technical Note No. 01/2013^17^, which provides prevention and control measures for infections caused by multidrug-resistant Enterobacterales.

### Extraction of bacterial DNA and investigation of target genes

Bacterial DNA was extracted from Müller-Hinton agar cultures using heat shock, as previously described^18^. To confirm the suitability of the extracted DNA for subsequent genotype analysis, the 16S rRNA gene was identified by the polymerase chain reaction (PCR)^19^. 

The presence of the target genes was also investigated using PCR. All reactions were carried out in a final volume of 50 μL, using 50-500 ng of extracted DNA. The PCR-amplified products were subjected to electrophoresis on 1% agarose gel and compared with a standard.

The *bla*_SHV_ and *bla*_CTX-M_ genes were investigated in isolates with positive phenotypic tests for the presence of ESBL, using specific primers. The thermocycling conditions consisted of an initial denaturation step at 94 ^o^C for 3 min, followed by 35 cycles of denaturation, annealing, and extension (1 min at 72 ^o^C) for each gene, and a final extension at 72 °C for 10 min.

To investigate the carbapenemase-encoding *bla*_OXA-48-like_, *bla*_NDM-1_, *bla*_KPC_, *bla*_SPM-1_, *bla*_VIM_, and *bla*_IMP_ genes, isolates showing phenotypic resistance to carbapenems were subjected to PCR consisting of an initial denaturation at 94 ^o^C for 3 min, followed by specific thermocycling conditions specific for each target gene. Reference strains were used to confirm the effectiveness of the target gene detection methods. 

### Statistical analysis

The samples were obtained using convenience sampling. Data were analyzed using descriptive statistics, with calculation of absolute and relative frequencies. Categorical variables are expressed as absolute numbers and percentages.

## RESULTS

Overall, 101 bacterial isolates were included in the study: 39 (38.6%) carbapenem-resistant *P. aeruginosa*, 37 (36.6%) ESBL-producing Enterobacterales, 15 (14.8%) *Klebsiella pneumoniae* with a positive phenotypic test for KPC, four (4.0%) *K. pneumoniae* with a positive phenotypic test for MβL, three (3.0%) carbapenem-resistant isolates of the CESP group (consisting of *Citrobacter freundii*, *Enterobacter* spp., *Serratia* spp., *Providencia* spp., *Morganella morganii,* and *Hafnia alvei*), and three (3.0%) carbapenem-resistant *K. pneumoniae*.

The isolates were collected from urine samples (31.7%; n=32), rectal swabs (16.8%; n=17), wound discharge (15.8%; n=16), bronchoalveolar lavage (11.9%; n=12), and other less common sites (23.8%; n=24). Regarding the distribution of isolates among hospital units, 53.5% (n=54) were from in-patient units, 20.8% (n=21) from the intensive care unit (ICU), 19.8% (n=20) from the emergency department, and 5.9% (n=6) were isolated at the surgical center and from out-patient units. Thirty-seven (36.6%) of the 101 isolates were obtained from surveillance cultures. 

### *In-vitro* activity of ceftolozane-tazobactam

The *in- vitro* activity of ceftolozane-tazobactam against each group of microorganisms selected in this study is shown in Table 1. The ESBL-producing Enterobacterales and CR *P. aeruginosa* isolates showed high susceptibility. A high resistance rate was observed in the KPC-producing *K. pneumoniae* isolates. Two CR *K. pneumoniae* isolates were resistant. All CR isolates of the CESP group and MβL-producing *K. pneumoniae* were resistant to ceftolozane-tazobactam.

**TABLE 1:** *In vitro* activity of ceftolozane-tazobactam.

	MIC frequency (%)												MIC interpretation (%)						MIC (mg/L)		
Group of microorganisms (N)													CLSI			BRCAST					
	<1	1	2	3	4	6	8	12	24	32	48	>256	S	I	R	S	I	R	MIC 50	MIC 90	Range
*Pseudomonas aeruginosa -* CR (39)	15.4	46.2	56.4	66.7	87.2	92.3	94.9			97.4		100	87.2	7.7	5.1	87.2		12.8	2	6	0.38 - >256
*Enterobacterales -* ESBL (37)	67.6	83.8	97.3									100	97.3		2.7	83.8		16.2	<1	2	0.125 - >256
*Klebsiella pneumoniae* - KPC (15)			13.3		20.0		26.7	33.3	53.3	60.0	86.7	100	13.3	6.6	80.0			100	24	>256	2,0 - >256
*Klebsiella pneumoniae* - MβL (4)												100			100			100	>256	>256	>256 - >256
*Klebsiella pneumoniae* - CR (3)	33.3								66.7		100		33.3		66.6	33.3		66.6	24	48	0.50 - 48
CESP group - CR (3)							33.3					100			100			100	>256	>256	8 - >256

MIC: minimum inhibitory concentration; S: susceptible; I: intermediate; R: resistant; MIC50 and MIC90 (mg/L): concentrations that inhibit 50% and 90% of the bacterial isolates, respectively. CLSI: Clinical and Laboratory Standards Institute; BRCAST: Brazilian Committee on Antimicrobial Susceptibility Testing. CR: resistance to at least one carbapenem; ESBL: extended-spectrum β-lactamase; KPC: *Klebsiella pneumoniae* carbapenemase; MβL: metallo-β-lactamase.

### *In-vitro* activity of ceftazidime-avibactam

Table 2 shows the *in-vitro* activity of ceftazidime-avibactam against each group of microorganisms selected in this study. All ESBL-producing Enterobacterales isolates were susceptible to ceftazidime-avibactam, showing the lowest MIC values compared with the other groups of microorganisms tested. A high susceptibility rate was observed for CR *P. aeruginosa*.

**TABLE 2:** *In vitro* activity of ceftazidime-avibactam.

	MIC frequency (%)										MIC interpretation (%)			MIC (mg/L)		
Group of microorganisms (N)											CLSI and BRCAST					
	<1	1	2	3	4	6	8	12	24	>256	S	I	R	MIC 50	MIC 90	Range
*Pseudomonas aeruginosa -* CR (39)	2.6	33.3	43.6	69.2	76.9	89.7	92.3	94.9	97.4	100	92.3		7.7	3	8	0.50 - >256
*Enterobacterales* - ESBL (37)	89.2	97.3		100							100			<1	1	0.125 - 3
*Klebsiella pneumoniae* - KPC (15)	26.7	86.7	93.3							100	93.3		6.6	1	2	0.38 - >256
*Klebsiella pneumoniae* - MβL (4)										100			100	>256	>256	>256 - >256
*Klebsiella pneumoniae* - CR (3)	33.3		66.7		100						100			2	4	0.75 - 4
CESP group - CR (3)		33.3			66.7			100			66.7		33.3	4	12	1 - 12

**MIC:** minimum inhibitory concentration; **S:** susceptible; **I:** intermediate; **R:** resistant; **MIC50 and MIC90 (mg/L):** concentrations that inhibit 50% and 90% of the bacterial isolates, respectively. **CLSI:** Clinical and Laboratory Standards Institute; **BRCAST:** Brazilian Committee on Antimicrobial Susceptibility Testing. **CR:** resistance to at least one carbapenem; **ESBL:** extended-spectrum β-lactamase; **KPC:** *Klebsiella pneumoniae* carbapenemase; **MβL:** metallo-β-lactamase.

The resistance rate of KPC-producing *K. pneumoniae* was low. All CR *K. pneumoniae* isolates were susceptible, whereas the MβL-producing *K. pneumoniae* isolates were resistant. Two bacterial isolates from the CESP group (*E. cloacae* and *S. marcescens*) were susceptible, and one was resistant to ceftazidime-avibactam.

### Phenotypic antimicrobial susceptibility

**FIGURE 1:**
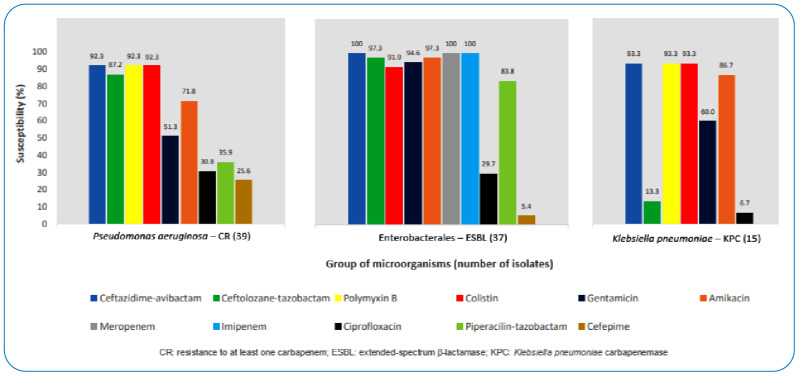
Susceptibility to ceftazidime-avibactam and ceftolozane-tazobactam compared to the other antimicrobials tested in the study.

Figure 1 summarizes the comparison of ceftazidime-avibactam and ceftolozane-tazobactam susceptibility profiles with the other antimicrobials tested in the different groups of microorganisms studied according to the CLSI breakpoints. 

### Genotypic resistance markers

The 16S rRNA gene was amplified in most of the isolates studied (97%, 98/101). The percentage of isolates positive for resistance genes was high (78.6%). Table 3 shows the distribution of the bacterial isolates according to the investigated genotypic resistance markers. All the tested isolates were negative for *bla*_SPM-1_, *bla*_OXA-48-like_, and *bla*_IMP_.

**TABLE 3:** Distribution of bacterial isolates according to genotypic resistance markers.

Phenotypic resistance	Species (N)	β-Lactamase genes	Isolates (n)	Isolates (%)
ESBL	*Escherichia coli* (27)	***bla***_CTX-M_	26	96.3
		***bla***_SHV_	17	63
	*Klebsiella pneumoniae* (7)	***bla***_CTX-M_	7	100
		***bla***_SHV_	6	85.7
	*Proteus mirabilis* (2)	***bla***_CTX-M_	2	100
	*Klebsiella ozaenae* (1)	***bla***_SHV_	1	100
		***bla***_CTX-M_	1	100
KPC	*Klebsiella pneumoniae* (15)	***bla***_KPC_	15	100
		***bla***_SHV_	14	93.3
		***bla***_CTX-M_	12	80
		***bla***_NDM-1_	1	6.7
		***bla***_VIM_	1	6.7
CR	*Pseudomonas aeruginosa* (36)	***bla***_CTX-M_	14	38.9
		***bla***_SHV_	5	13.9
		***bla***_KPC_	2	5.6
		***bla***_VIM_	1	2.8
	*Klebsiella pneumoniae* (3)	***bla***_CTX-M_	3	100
		***bla***_SHV_	3	100
	*Klebsiella pneumoniae* (3)	***bla***_NDM-1_	1	33.3
	*Enterobacter cloacae* (2)	***bla***_CTX-M_	1	50
	*Serratia marcescens* (1)	***bla***_KPC_	1	100
MβL	*Klebsiella pneumoniae* (4)	***bla***_SHV_	4	100
		***bla***_NDM-1_	3	75

**ESBL:** extended-spectrum β-lactamase; **KPC:** *Klebsiella pneumoniae* carbapenemase; **CR:** resistance to at least one carbapenem; **MβL:** metallo-β-lactamase.

Coexistence of resistance genes was observed in 44 (44.9%) isolates. The most prevalent combinations were *bla*_KPC_+*bla*_CTX-M_+*bla*_SHV_ in KPC-producing *K. pneumoniae*, and *bla*_CTX-M_+*bla*_SHV_ in ESBL-producing *Escherichia coli*. Most isolates with phenotypic resistance to ceftazidime-avibactam and ceftolozane-tazobactam concomitantly carried two or more β-lactamase-encoding genes (Table 4). The *bla*_CTX-M_, *bla*_SHV_, and *bla*_KPC_ were most frequently detected in isolates resistant to ceftolozane-tazobactam, whereas *bla*_SHV_, *bla*_CTX-M_, and *bla*_NDM-1_ were most frequently detected in isolates resistant to ceftazidime-avibactam. 

**TABLE 4:** Presence of β-lactamase-encoding genes and phenotypic susceptibility to ceftazidime-avibactam (C/A) and ceftolozane-tazobactam (C/T) in the isolates studied.

Phenotypic resistance	Species	Isolates (n)	β-Lactamase genes	Phenotype	
				C/A	C/T
ESBL	*Escherichia coli*	15	***bla***_CTX-M_ ***, bla***_SHV_	S	S
		10	***bla***_CTX-M_	S	S
		1	***bla***_CTX-M_ **, *bla***_SHV_	S	R
		1	***bla***_SHV_	S	S
	*Klebsiella pneumoniae*	5	***bla***_CTX-M_	S	S
		1	***bla***_CTX-M_ **, *bla***_SHV_	S	R
		1	***bla***_CTX-M_	S	R
	*Proteus mirabilis*	2	***bla***_CTX-M_	S	R
	*Klebsiella ozaenae*	1	***bla***_CTX-M_ **, *bla***_SHV_	S	R
KPC	*Klebsiella pneumoniae*	9	***bla***_KPC_ ***, bla***_CTX-M_ ***, bla***_SHV_	S	R
		2	***bla***_KPC_ ***, bla***_SHV_	S	R
		1	***bla***_KPC_ ***, bla***_CTX-M_ ***, bla***_SHV_ ***, bla***_NDM-1_	S	R
		1	***bla***_KPC,_ ***bla***_CTX-M_ ***, bla***_SHV_ ***, bla***_VIM_	S	R
		1	***bla***_KPC_ ***, bla***_CTX-M_	S	R
		1	***bla***_KPC_ ***, bla***_SHV_	R	R
CR	*Pseudomonas aeruginosa*	7	***bla***_CTX-M_	S	S
		2	***bla***_CTX-M_ **, *bla***_SHV_	S	S
		1	***bla***_KPC_ ***, bla***_CTX-M_	S	S
		1	***bla***_KPC_ ***, bla***_CTX-M_ ***, bla***_SHV_	S	S
		1	***bla***_SHV_	S	S
		1	***bla***_VIM_	S	S
		1	***bla***_CTX-M_	R	S
		1	***bla***_CTX-M_	S	R
		1	***bla***_CTX-M_ **, *bla***_SHV_	R	R
	*Klebsiella pneumoniae*	2	***bla***_CTX-M_ **, *bla***_SHV_	S	R
		1	***bla***_CTX-M_ **, *bla***_SHV_ ***, bla***_NDM-1_	S	S
	*Enterobacter cloacae*	1	***bla***_CTX-M_	R	R
	*Serratia marcescens*	1	***bla***_KPC_	S	R
MβL	*Klebsiella pneumoniae*	3	***bla***_SHV_ ***, bla***_NDM-1_	R	R
		1	***bla***_SHV_	R	R

**ESBL:** extended-spectrum β-lactamase; **KPC:** *Klebsiella pneumoniae* carbapenemase; **CR:** resistance to at least one carbapenem; **MβL:** metallo-β-lactamase. **C/A:** ceftazidime-avibactam; **C/T:** ceftolozane-tazobactam. **R:** resistant; **S:** susceptible.

## DISCUSSION

The increasing incidence of bacterial strains isolated from clinical samples that produce carbapenemases, enzymes capable of inactivating carbapenems, and most β-lactams^20^ represents the greatest challenge of antibiotic therapy in recent years^2^ ^,^ ^6^. Enterobacterales and *P. aeruginosa* are the main causative agents of severe infections associated with antibiotic resistance resulting from chromosomal mutations and the transfer of plasmid-mediated resistance^21^. Such clinical isolates commonly produce carbapenemases and exhibit multidrug resistance and pan-resistance phenotypes^22^. 

Studies have been conducted in different countries to evaluate *in-vitro* susceptibility to ceftazidime-avibactam and ceftolozane-tazobactam, and bacterial resistance to these antimicrobial agents has been reported in hospitalized patients with or without previous treatment^16^. Furthermore, the combination of resistance mechanisms can significantly increase the MIC of ceftazidime-avibactam and ceftolozane-tazobactam^16^ ^,^ ^23^.

The ability of ceftazidime-avibactam to inhibit KPC-type β-lactamases has attracted global interest. In China, Cui et al. evaluated 347 KPC-producing *K. pneumoniae* isolates collected from patients without previous treatment with only 12 (3.5%) isolates showing reduced susceptibility to ceftazidime-avibactam^24^. In Brazil, Rossi et al. found that among 30 selected *K. pneumoniae* isolates that were not susceptible to meropenem and positive for *bla*_KPC_, only one (3.3%) was resistant to ceftazidime combined with avibactam^9^. Similarly, in our study, the presence of *bla*_KPC_ did not influence ceftazidime-avibactam susceptibility, and the resistance rate observed (6.6%; 1/15) was consistent with the global surveillance results of carbapenem-resistant and *bla*_KPC_-carrying *K. pneumoniae* ^10^.

Jonge et al. characterized the *in vitro* activity of ceftazidime-avibactam against 961 meropenem-non-susceptible Enterobacterales isolates from Europe, Asia, Latin America, and the Middle East using a global antimicrobial resistance surveillance program. The authors evaluated 145 MβL-producing isolates and detected a ceftazidime-avibactam resistance rate of 96.6%^10^. Similarly, all MβL-producing *K. pneumoniae* isolates in this study were resistant to ceftazidime-avibactam. Thus, ceftazidime-avibactam is a potent agent against carbapenem-resistant Enterobacterales, except for isolates in which resistance is mediated by MβL.

According to international reports, the resistance rates of *P. aeruginosa* to ceftazidime-avibactam are higher than those reported for Enterobacterales, ranging from 2.9 to 18%, whereas these rates can reach 50% in isolates resistant to carbapenems^16^. Although this study investigated carbapenem-resistant *P. aeruginosa* isolates, the rate of ceftazidime-avibactam resistance was low (7.7%; 3/39). This might be related to the absence of carbapenemase-encoding genes in most of the isolates investigated, suggesting that the detected phenotypic resistance to carbapenems is associated with other pseudomonal resistance mechanisms not analyzed here/in this study.

Studies have reported that bacteremia caused by ESBL-producing Enterobacterales is associated with higher rates of treatment failure and patient mortality when compared to bacteremia caused by non ESBL-producing strains^1^ ^,^ ^25^. Various authors have emphasized that ESBL-producing Enterobacterales strains are not associated with resistance to ceftazidime-avibactam^6^ ^,^ ^7^ ^,^ ^10^ ^,^ ^15^ ^,^ ^25^ ^,^ ^26^, which was also demonstrated in our study.

López-Calleja et al. analyzed the multidrug-resistant and extensively drug-resistant non-MβL-producing *P. aeruginosa* isolates collected in Spain and reported 92.2% susceptibility to ceftolozane-tazobactam, which was the second most active antimicrobial agent after colistin^27^; this was also observed in our study (87.2%). In particular, we did not identify *bla*_NDM-1_ or *bla*_IMP_ genes in carbapenem-resistant *P. aeruginosa* isolates, and the *bla*_VIM_ gene detected in only one isolate was not associated with the ceftolozane-tazobactam resistance phenotype. In contrast, Teo et al. highlighted the importance of geographic variation in antimicrobial activity. The authors reported much lower susceptibility rates of *P. aeruginosa* to ceftolozane-tazobactam (37.9%), which was associated with the presence of MβL, compatible with local molecular epidemiology^28^.

Tuon et al. evaluated 673 GNB isolates collected from different Brazilian centers and found rates of *in-vitro* susceptibility to ceftolozane-tazobactam ranging from 40.4% to 94.9%. The susceptibility rate of *K. pneumoniae* to ceftolozane-tazobactam was low (40.4%) because of the high incidence of KPC-type carbapenemases in Brazil, an enzyme that catalyzes the hydrolysis of ceftolozane^29^. In our study, the susceptibility rate of KPC-producing *K. pneumoniae* to ceftolozane-tazobactam was even lower (13.3%). This finding might be explained by the identification of *bla*_KPC_ gene in all isolates tested and the concomitant presence of ESBL-encoding genes. Additionally, two isolates carrying more than one carbapenemase- and ESBL-encoding gene (*bla*_KPC_ *+bla*_CTX-M_ *+bla*_SHV_ *+bla*_NDM-1_ and *bla*_KPC_+*bla*_CTX-M_ *+bla*_SHV_ *+bla*_VIM_) were identified in association with ceftolozane-tazobactam resistance phenotypes. Regarding MβL-producing *K. pneumoniae*, all isolates were resistant to ceftolozane-tazobactam and most of them carried *bla*_NDM-1_, suggesting that this agent should be used with caution in empirical therapies.

In contrast, we found high rates of *in-vitro* ceftolozane-tazobactam susceptibility among ESBL-producing Enterobacterales. This finding in consistent with a study that evaluated 21,952 Enterobacterales isolates from 51 countries and found that ceftolozane-tazobactam inhibited 82.4% of ESBL-producing isolates^15^.

This study had some limitations. The number of isolates evaluated was relatively small, and the study was conducted at a single hospital. However, the study used clinical isolates selected in recent years to better reflect the current epidemiological scenario. Therefore, we recommend multicenter studies using a phenotypic and genotypic approach and a greater number of multidrug-resistant isolates for better understanding of the local molecular epidemiology and for the detection of resistance to ceftazidime-avibactam and ceftolozane-tazobactam in different regions of Brazil. 

In conclusion, we found high susceptibility rates to ceftazidime-avibactam and ceftolozane-tazobactam among ESBL-producing Enterobacterales and carbapenem-resistant *Pseudomonas aeruginosa*. In contrast, carbapenemase-producing *Klebsiella pneumoniae* exhibited high resistance to ceftolozane-tazobactam but high susceptibility to ceftazidime-avibactam. Our results obtained *in-vitro* confirmed that ceftazidime-avibactam and ceftolozane-tazobactam were active against microorganisms with β-lactam resistance phenotypes, except when resistance was mediated by metallo-β-lactamases. Additionally, most ceftazidime-avibactam- and ceftolozane-tazobactam-resistant isolates concomitantly carried two or more β-lactamase-encoding genes.

## References

[1] Friedman ND, Temkin E, Carmeli Y (2016). The negative impact of antibiotic resistance. Clin Microbiol Infect.

[2] Sekar R, Srivani S, Kalyanaraman N, Thenmozhi P, Amudhan M, Lallitha S (2019). New Delhi Metallo-β-lactamase and other mechanisms of carbapenemases among Enterobacteriaceae in rural South India. J Glob Antimicrob Resist.

[3] Wu C, Wang Y, Shi X, Wang S, Ren H, Shen Z (2018). Rapid rise of the ESBL and mcr-1 genes in Escherichia coli of chicken origin in China, 2008-2014. Emerg Microbes Infect.

[4] Melo LC, Oresco C, Leigue L, Netto HM, Melville PA, Benites NR (2018). Prevalence and molecular features of ESBL/pAmpC-producing Enterobacteriaceae in healthy and diseased companion animals in Brazil. Vet Microbiol.

[5] World Health Organization (2017). Global priority list of antibiotic-resistant bacteria to guide research, discovery, and development of new antibiotics.

[6] Alatoom A Elsayed H, Lawlor K AbdelWareth L, El-Lababidi Cardona L (2017). Comparison of antimicrobial activity between ceftolozane - tazobactam and ceftazidime - avibactam against multidrug-resistant isolates of Escherichia coli, Klebsiella pneumoniae, and Pseudomonas aeruginosa. Int J Infect Dis.

[7] Tuon FF, Rocha JL, Formigoni-Pinto MR (2017). Pharmacological aspects and spectrum of action of ceftazidime-avibactam: a systematic review. Infection.

[8] BRASIL (2018). Agência Nacional de Vigilância Sanitária. Parecer público de avaliação de medicamento-aprovação.

[9] Rossi F, Cury AP, Franco MRG, Testa R, Nichols WW (2017). The in vitro activity of ceftazidime-avibactam against 417 Gram-negative bacilli collected in 2014 and 2015 at a teaching hospital in São Paulo, Brazil. Braz J Infect Dis.

[10] Jonge BLM, Karlowsky JA, Kazmierczak KM, Biedenbach DJ, Sahm DF, Nichols WW (2016). In vitro susceptibility to ceftazidime-avibactam of carbapenem-nonsusceptible Enterobacteriaceae isolates collected during the INFORM global surveillance study (2012 to 2014). Antimicrob Agents Chemother.

[11] Garcia-Fernandez S, García-Castilho M, Melo-Cristino J, Pinto MF, Gonçalves E, Alves V (2019). In vitro activity of ceftolozane-tazobactam against Enterobacterales and Pseudomonas aeruginosa causing urinary, intra-abdominal and lower respiratory tract infections in intensive care units in Portugal: the STEP multicentre study. Int J Antimicrob Agents.

[12] Carvalhães CG, Castanheira M, Sader HS, Flamm RK, Shortridge D (2019). Antimicrobial activity of ceftolozane-tazobactam tested against gram-negative contemporary (2015-2017) isolates from hospitalized patients with pneumonia in US medical centers. Diagn Microbiol and Infect Dis.

[13] Gherardi G, Linardos G, Pompilio A, Fiscarelli E, Di Bonaventura D (2019). Evaluation of in vitro activity of ceftolozane-tazobactam compared to other antimicrobial agents against Pseudomonas aeruginosa isolates from cystic fibrosis patients. Diagn Microbiol and Infect Dis.

[14] Gómez-Junyent J, Benavent E, Sierra Y, El Haj C, Soldevila L, Torrejón B (2019). Efficacy of ceftolozane/tazobactam, alone and in combination with colistin, against multidrug-resistant Pseudomonas aeruginosa in an in vitro biofilm pharmacodynamic model. Int J Antimicrob Agents.

[15] Karlowsky JA, Kazmiercza KM, Young K, Motyl MR, Sahm DF (2019). In vitro activity of ceftolozane/tazobactam against phenotypically defined extended-spectrum β-lactamase (ESBL)-positive isolates of Escherichia coli and Klebsiella pneumoniae isolated from hospitalized patients (SMART 2016). Diagn Microbiol and Infect Dis.

[16] Wang Y, Wang J, Wang R, Cai Y (2020). Resistance to ceftazidime-avibactam and underlying mechanisms. J Glob Antimicrob Resist.

[17] ANVISA (2013). Nota Técnica n 01/2013: Medidas de prevenção e controle de infecções por enterobactérias multirresistentes.

[18] Kobs VC, Augustini FJ, Bobrowicz TA, Ferreira LE, Deglmann RC, Westphal GA (2016). The role of the genetic elements bla oxa and ISAba1 in the Acinetobacter calcoaceticus-Acinetobacter baumannii complex in carbapenem resistance in the hospital setting. Rev Soc Bras Med Trop.

[19] Eden PA, Schmidt TM, Blakemore RP, Pace NR (1991). Phylogenetic analysis of aquaspirillum magnetotacticum using polymerase chain reaction-amplified 16S rRNA-specific DNA. Inter Journal Sys Bacteriol.

[20] Karam G, Chastre J, Wilcox MH, Vincent JL (2016). Antibiotic strategies in the era of multidrug resistance. Crit Care.

[21] Van Duin D, Doi Y (2016). The global epidemiology of carbapenemase-producing Enterobacteriaceae. Virulence.

[22] Magiorakos AP, Srinivasan A, Carey RB, Carmeli Y, Falagas ME, Giske CG (2012). Multidrug-resistant, extensively drug-resistant and pandrug-resistant bacteria: An international expert proposal for interim standard definitions for acquired resistance. Clin Microbiol Infect.

[23] Schillaci D, Spano V, Parrino B, Carbone A, Montalbano A, Barraja P (2017). Pharmaceutical approaches to target antibiotic resistance mechanisms. J Med Chem.

[24] Cui X, Shan B, Zhang X, Qu F, Jia W, Huang B (2020). Reduced ceftazidime-avibactam susceptibility in KPC-producing Klebsiella pneumoniae from patients without ceftazidime-avibactam use history - A Multicenter Study in China. Front Microbiol.

[25] Sah BS, Aryal M, Bhargava D, Siddique A (2017). Drug resistance pattern of bacterial pathogens of Enterobacteriaceae family. TUJM.

[26] Castanheira M, Doyle TB, Mendes RE, Sader HS (2019). Comparative activities of ceftazidime-avibactam and ceftolozane-tazobactam against Enterobacteriaceae isolates producing extended-spectrum β - lactamases from U.S. hospitals. Antimicrob Agents Chemother.

[27] Lopéz-Calleja AI, Morales EM, Medina RN, Esgueva MF, Pareja JS, Gárcia-Lechuz JM (2019). Antimicrobial activity of ceftolozane-tazobactam against multidrug-resistant and extensively drugresistant Pseudomonas aeruginosa clinical isolates from a Spanish hospital. Rev Esp Quimioter.

[28] Qi-Min Teo J, Lim JC, Tang CY, Lee SJY, Tan SH, Sim JHC (2021). Ceftolozane/tazobactam resistance and mechanisms in carbapenem-nonsusceptible Pseudomonas aeruginosa. mSphere.

[29] Tuon FF, Cieslinski J, Rodrigues SS, Serra FB, De Paula MDN (2020). Evaluation of in vitro activity of ceftolozane-tazobactam against recent clinical bacterial isolates from Brazil - the EM200 study. Braz J Infect Dis.

